# Developmental Exposure to 2,2′,4,4′-Tetrabromodiphenyl Ether Permanently Alters Blood-Liver Balance of Lipids in Male Mice

**DOI:** 10.3389/fendo.2018.00548

**Published:** 2018-09-20

**Authors:** Ahmed Khalil, Sebnem E. Cevik, Stephanie Hung, Sridurgadevi Kolla, Monika A. Roy, Alexander Suvorov

**Affiliations:** ^1^Department of Environmental Health Sciences, University of Massachusetts Amherst, Amherst, MA, United States; ^2^Medical Biotechnology Department, Genetic Engineering & Biotechnology Research Institute, City of Scientific Research & Technological Applications, Alexandria, Egypt

**Keywords:** polybrominated diphenyl ether, *Cd36*, fatty acid, triglyceride, metabolism, rodent, ribosome, NAFLD

## Abstract

Polybrominated diphenyl ethers (PBDEs) were used as flame-retardant additives starting 1965 and were recently withdrawn from commerce in North America and Europe. Approximately 1/5 of the total U.S. population were born when environmental concentrations of PBDE plateaued at their maximum. Accumulating evidence suggests that developmental exposures to PBDE may result in long-lasting programming of liver metabolism. In this study, CD-1 mice were exposed prenatally or neonatally to 1 mg/kg body weight of 2,2′,4,4′-tetrabromodiphenyl ether (BDE-47), and changes in liver histology, transcriptome, and liver-blood balance of triglycerides were analyzed in 10 months old male offspring. In both exposure groups, long-term reprogramming of lipid metabolism was observed, including increased liver triglycerides and decreased blood triglycerides, and altered expression of metabolic genes in the liver. Significant upregulation of lipid influx transporter *Cd36* 2.3- and 5.7-fold in pre- and neonatal exposure groups, respectively was identified as a potential mechanism of blood/liver imbalance of triglycerides. Analysis of our and previously published all-genome gene expression data identified changes in expression of ribosomal protein genes as a transcriptomic signature of PBDE exposure. Further comparison of our new data and published data demonstrate that low doses (0.2 mg/kg body weight) of PBDE induce long-lasting up-regulation of ribosomal genes, suppression of *Cd36* in liver and increase circulating triglycerides in blood, while moderated doses (≥1 mg/kg body weight) produce opposite long-lasting effects. To conclude, this study shows that an environmentally relevant developmental exposures to BDE-47 permanently alter lipid uptake and accumulation in the liver, with low and moderate doses having opposite effect on liver transcriptomics and triglyceride balance. Similar effects of pre- and neonatal exposures point at hepatocyte maturation as a sensitive window of the liver metabolism programming. These results suggest that PBDE exposure may be an important factor increasing risks of cardio-vascular disease and non-alcoholic fatty liver disease via modulation of liver/blood balance of lipids. The translational relevance of these findings for human remain to be studied.

## Introduction

Polybrominated diphenyl ethers (PBDEs) are a group of ubiquitous and persistent chemical compounds that include 209 congeners. PBDEs are highly lipophilic xenobiotics (1, 2) with a half-life of 1.8–6.5 years in human tissues (3). PBDEs persist in the environment due to pollution by industrial chemicals and biosynthesis in natural ecosystems. The manufacturing of commercial products containing PBDEs began in 1965 (4) and synthetic PBDEs were widely used as flame-retardant additives in a range of products, including building materials, electronics, furnishings, motor vehicles, airplanes, plastics, polyurethane foams, and baby pajamas. Production of PBDEs was banned in Europe in 2003 because of concerning toxicological evidence (5). In the US PBDEs were voluntarily withdrawn from commercial use by industry by 2013 (6). Before these decisions were made PBDEs increased exponentially in human blood, breast milk and tissues (7), including fetal tissues (8) over a 30 years period. In Europe, the discontinued use of PBDEs led to a decrease in environmental concentrations in the recent years. However, an epidemiological study of 1,253 women in California suggests that PBDE concentrations continue to rise in North America (9). This on-going persistence of PBDE exposure in humans can be attributed to many factors, including, the prevalence of PBDE in waste and recycling sites, indoor use of products containing PBDEs (10), global circulation of PBDE toward the Northern Hemisphere, biosynthesis of PBDE by microflora of the marine environment including γ-proteobacteria (11), and endosymbiotic microflora of benthic sponges (12–15) and high bioaccumulation and bioconcentration of PBDEs in food chains (16). Younger generations of Americans that were exposed *in utero* and during early postnatal life to the highest environmental doses of PBDE account for approximately one-fifth of the US population (17, 18). The long-term consequences of developmental exposures to PBDE for this population are not well-understood.

In the general population, exposures to highly lipophilic environmental xenobiotics usually peak during fetal and early postnatal development, due to the active transfer of lipophilic compounds from the mothers' storage depots through cord blood and breast milk (19, 20). Additional factors that lead to an increased exposure during the early postnatal period are the ingestion of PBDE-containing household dust by toddlers (21) and high food intake per kilogram of body weight by toddlers (22). As a result, breastfeeding toddlers have several-fold higher plasma levels of PBDEs than their mothers (23). Thus, PBDE exposure is highest during early stages of development (*in utero* and early postnatal) when biological plasticity is high and even modest exposures to environmental stressors may result in dramatic and long-term health effects (24–26).

The ability of chemical compounds to disrupt lipid metabolism in liver has been known for many years (27). The term “metabolic disruptor” was suggested recently by a group of experts to characterize the growing list of environmental compounds that are able to alter metabolism in the mammalian organism (26). Another term, “toxicant-associated steatohepatitis” (TASH) was coined (28) to characterize cases of fatty-liver disease in highly exposed chemical workers. The ability of chemical compounds to produce long lasting changes in lipid metabolism following developmental short-term exposures is poorly understood, although this type of exposure is relevant for many lipophilic environmental xenobiotics. In our recent study, we reported that mice exposed to 0.2 mg/kg body weight of 2,2′,4,4′-tetrabromodiphenyl ether (BDE-47), the most prevalent PBDE congener in human samples, from gestation day 8 (GD8) till postnatal day 21 (PND21), develop long lasting suppression of fatty acid (FA) translocase *Cd36* in hepatocytes (18), a membrane receptor responsible for FA uptake. Changes in expression of this gene were concordant with increased blood triglycerides in exposed animals (18). Increased level of blood lipids is the primary risk factor for heart attack, which is the most common cause of mortality in the developed world, with more than 700,000 deaths attributed to the disease in the US annually (29). Exposure of significant part of the general population to PBDE during early stages of development together with the ability of PBDE to program lipid metabolism in a mammalian organism by early life exposures raises significant concerns about metabolic health of young Americans and suggests a need for additional studies, to examine the relations between early life PBDE exposures and permanent changes in liver metabolism.

In this study we analyze developmental windows sensitive to liver metabolism programming by PBDE using a mouse model. We report that exposures to 1 mg/kg body weight of BDE-47 during prenatal or neonatal windows produce similar changes in 10 months old mice. These changes include altered liver transcriptome, including increased expression of *Cd36*, altered balance of triglycerides between blood and liver and hepatic steatosis-like phenotype. All these effects are opposite in direction in comparison with effects observed in our previous study (18), where mice were exposed to 0.2 mg/kg body weight of BDE-47 perinatally, suggesting complex dose-response relationships for developmental programming of liver metabolism by PBDEs.

## Materials and methods

### Animals and treatment

Timed pregnant CD-1 mice were obtained from Charles River Laboratories (Kingston, NY, USA) at pregnancy day 6 and housed in a temperature (23 ± 2°C)- and humidity (40 ± 10%)-controlled environment, with a 12-h light/dark cycle, and food and water available *ad libitum*. The dams were assigned to one of three treatment groups (*n* = *10* per group) based on weight match. The control group was exposed to vehicle—tocopherol-stripped corn oil (MP Biomedicals, Solon, OH) from pregnancy day 8 till postpartum day 21. The prenatal exposure group received 1 mg/ml solution of BDE-47 (AccuStandard, Inc., New Haven, CT; 100% purity) in tocopherol-stripped corn oil from pregnancy day 8 through delivery and then vehicle only from the day of delivery till postpartum day 21. Neonatal exposure group received only vehicle from gestational day 8 till delivery and was then exposed to 1 mg/ml solution of BDE-47 in tocopherol-stripped corn oil through postpartum day 21. Either vehicle or BDE-47 solution was orally administered daily via micropipette in a volume of 1 μl/gram body weight (BW) resulting in exposures to 1 mg/kg BW/day during pre- or neonatal periods. This method of exposure is routinely used in our laboratory as a substitution of oral gavage, which induces a significant stress response by the endocrine system, which may interfere with the evaluation of endpoints of interest (30). The dams delivered naturally, and the litters were not culled to maintain consistency of nutrient distribution among the same number of fetuses/pups at pre- and postnatal periods, and to avoid catch-up growth (31). The dams and pups were kept together until weaning on postnatal day (PND) 21, when the male and female pups were separated. One randomly selected male pup per litter was euthanized using cervical dislocation on PND300 between 9 and 11 a.m. following 2 h of fasting. Tissue samples were collected immediately upon euthanasia. Blood samples were centrifuged at 3,000 g for 10 min and serum was collected and stored at -80°C. Liver was snap-frozen in liquid nitrogen and stored at -80°C. Only male pups were used for further analysis to avoid the interaction of measured health outcomes with hormonal fluctuations due to estrus cycle. Other male and female pups were used in a different study. All procedures met the guidelines of the National Institutes of Health Guide for the Care and Use of Laboratory Animals and this study was approved by the Institutional Animal Care and Use Committee at University of Massachusetts, Amherst.

### RNA extraction and sequencing

Total RNA was isolated from liver samples using TRIzol reagent (Invitrogen) and quantified using a NanoDrop 1000 instrument (Thermo Fisher Scientific, Wilmington, DE). RNA quality was assessed using Agilent 2100 Bioanalyzer (Agilent Technologies, Santa Clara, CA). Samples of RNA isolated from liver tissue with integrity values >9 were used for library preparation. RS-122-2101-TruSeq® Stranded mRNA LT-SetA kit (Illumina, San-Diego, CA) was used to isolate intact poly(A)+ RNA from 4 μg of total RNA and to construct strand-specific libraries with multiplexing indexes. The quality and purity of the libraries were assessed using the Agilent 2100 Bioanalyzer. The concentration of the libraries was measured using Qubit 3.0 fluorometer (Life Technologies, Carlsbad, CA). High-throughput sequencing was performed using NextSeq500 sequencing system (Illumina, San-Diego, CA) in the Genomic Resource Laboratory of the University of Massachusetts, Amherst. cDNA libraries were single-end sequenced in 76 cycles using a NextSeq 500 Kit v2 (FC-404-2005, Illumina, San-Diego, CA) in one multiplex run (3–4 samples per exposure group). All sequencing data were uploaded to the GEO public repository and GEO were assigned series accession number GSE115143.

### Analysis of mouse RNA-seq data

Read filtering, trimming and de-multiplexing were performed using the BaseSpace cloud computing service supported by Illumina (https://basespace.illumina.com/home/index). Furthermore, the preprocessed reads were mapped to the reference mouse genome (MM10) using TopHat 2 aligner (32). Aligned reads were then used for assembly of novel transcripts with Cufflinks 2.1.1 and differential expression of novel and reference transcripts with Cuffdiff 2.1.1 (33). Differential expression datasets were further used for gene set enrichment analysis (GSEA, www.broadinstitute.org/gsea). This approach is particularly effective for the identification of biologically significant changes in gene expression that are associated with relatively small effects across multiple members of a gene set (34). The details of the method and statistical approaches used by GSEA are described elsewhere (35, 36). We used GSEA against the Hallmark, KEGG and Reactome collections of datasets. Short-lists of significantly differentially expressed genes were identified by applying thresholds of 2-fold differential expression and false discovery rate (FDR) *q* ≤ 0.05. These lists were analyzed using DAVID Functional Annotation Clustering tool (37) and Disease and BioFunctions tool of Ingenuity Pathway Analysis (38) with default settings. To explore further liver lipid metabolism mechanisms affected by BDE-47 we inspected changes in expression of genes, that encode key enzymes of lipid trafficking, de-novo synthesis and/or disposal (39, 40). The following genes were selected for analysis (see Supplemental File 1): fatty acid uptake proteins—fatty acid translocase (FAT; gene *Cd36*) and fatty acid uptake proteins (FATP; *Slc27a* genes); de-novo synthesis of fatty acids—acetyl-CoA carboxylase (ACC; genes *Acaca* and *Acacb*) and fatty acid synthase (FAS; gene *Fasn*); synthesis of triglycerides—glycerol-3-phosphate acyltransferase (GPAT; gene *Gpam*), 1-acylglycerol-3-phosphate O-acyltransferase (AGPAT; *Agpat* genes), diacylglycerol O-acyltransferase (DGAT; *Dgat* genes), lipin (LIPIN; *Lpin* genes), mannoside acetylglucosaminyltransferase (MGAT; *Mgat1* genes), and adiponutrin (gene *Pnpla3*); oxidation of fatty acids—acyl-CoA synthetase (ACS; *Acsl, Acsm*, and *Acss* genes), carnitine palmitoyltransferase (CPT; genes *Cpt1a, Cpt1b, Cpt1c, Cpt2*), adipose triglyceride lipase (ATGL, gene *Pnpla2*), and 3-hydroxy-3-methylglutaryl-coenzyme A synthase (HMG-CoA; *Hmgcs* genes); very low density lipoprotein (VLDL) secretion—apolipoprotein B (gene *Apob*), microsomal triglyceride transfer protein (MTP, gene *Mttp*), carboxylesterase (CES; genes *Ces1d, Ces1g*, and *Ces3a*), and cell death-inducing DFF-45-like effector B (gene *Cideb*).

### RT-qPCR

The RNA-seq results were validated using RT-qPCR for selected genes: *Cd36, Abcd2, Prelid2, Apoa4, Fabp4, Fgl1, Gdpd3*, and *Hao2*. These gene were selected based on the following criteria (1) they were significantly regulated in both exposure groups based on RNA-seq results and (2) they encode proteins important for liver metabolic function. RT-qPCR was done using independent set of samples, six per exposure group. Total RNA was purified of genomic DNA contamination using DNase (RQ1 RNAse-free DNAse, Cat. # M610A, Promega, Madison, WI), and reverse transcribed using the High Capacity cDNA Reverse Transcription Kit (Cat.# 4368814, Applied Biosystems, Vilnus, Lithuania). Using free online software Primer3Plus (http://primer3plus.com/cgi-bin/dev/primer3plus.cgi), forward and reverse primers were designed to anneal different exons spanning long intron (Table 1). The housekeeping gene (*B2m*) was selected from genes that were not regulated in our RNA-seq dataset, with the consideration of its ubiquitous presence in different cell types. A triplicate of 5 μl real-time PCR reactions, each containing iTaq Universal SYBR Green Supermix (Cat 172-5124, BioRad), primers, and cDNA template were loaded onto a 384-well plate and run through 40 cycles on a CFX384 real time cycler (Bio-Rad Laboratories, Inc). The data were analyzed using the manufacturer's CFX manager software, version 3.1. Relative quantification was determined using the ΔΔCq method (41).

**Table 1:** Quantitative reverse transcription polymerase chain reaction (RT-qPCR) validation of RNA-seq genes for selected differentially expressed genes.

		**Fold change**			
**Gene**	**qPCR primers**	**Prenatal exposure**		**Postnatal exposure**	
		**RNA-seq**	**qPCR**	**RNA-seq**	**qPCR**
Cd36	TGTGTTTGGAGGCATTCTCA TTTGAAAGCAGTGGTTCCTTC	2.25	2.01	5.58	4.99
Abcd2	TGTGGAGCAGCTGTGGACTA CATAGCCTGCTTTGGACCAT	11.00	9.76	5.90	5.37
Prelid2	TGTTCCAGTACCCCTTCGAG TTCCACAGTTTTTACAGAGATGACA	−5.28	−6.11	−5.13	−6.20
Apoa4	AGTGAGGAGCCCAGGATGTT CACCTGGTCCGAAGTGACCT	−3.48	−2.98	−3.81	−3.45
Fabp4	AATGTGTGATGCCTTTGTGG CACTTTCCTTGTGGCAAAGC	−6.68	−5.34	−6.06	−7.01
Fgl1	GTGGATGGACTGAGCCTAGC TTCCCATTCTTCCCACTGAG	−2.41	−2.55	−6.59	−5.17
Gdpd3	CCTTTTGTCTCCATCCCTGA CCACAGCGAAATGGGAAGTA	16.91	11.39	6.23	5.86
Hao2	GAGGCAGCTTGATGAGGTTC CCCACCATCCATGTACACTTC	4.86	5.12	7.73	6.03
B2m	CCGGCCTGTATGCTATCCAG TGTTCGGCTTCCCATTCTCC				Housekeeping gene

### Protein expression of Cd36

Expression of Cd36 was analyzed by western blotting in liver samples of male offspring euthanized on PND300. Liver samples were lysed in T-PER tissue protein extraction buffer (ThermoFisher Scientific, Cat. # 78510) containing protease and phosphatase inhibitors cocktail (ThermoFisher Scientific, Cat. # 78442). A microplate-based BCA Protein Assay Kit (ThermoFisher Scientific, Cat. # 23227) was then used to determine protein concentrations. Western blot analyses were performed after separating the proteins on 4–20% SDS-PAGE gels (Bio-Rad, Cat. # 456-1094) and transferring them onto a PVDF membrane (0.2 μm) under wet conditions using a Biorad mini trans-blot cell. Anti-Cd36 (1:2000, Cell Signaling, Cat. #14347) and anti-Actin (1:2000, Cell Signaling, Cat. # 4970) primary antibodies were used at indicated dilutions. Proteins were visualized using secondary antibody conjugated with HRP (1:5000, Abcam, Cat. # ab6721), and Pierce ECL enhanced chemiluminesence reagent (ThermoFisher Scientific, Cat. # 32106). Western blot densitometry was quantified using Image Studio Lite Ver 5.2 software.

### Histological analysis

Oil Red O staining was performed in accordance with the protocol for frozen tissue described elsewhere [IHS 42)]. Liver samples were sectioned at 10 μm for lipid staining. Sections were fixed in 10% neutral buffered formalin for 10 min, rinsed with water, and immersed in 100% propylene glycol for 5 min. Slides were stained with Oil Red O solution (Sigma) for 10 min at 60°C, and placed in 85% propylene glycol for 5 min followed by a rinse in distilled water. Slides were counterstained with Harris' hematoxylin for 30 sec, washed in running tap water and coverslipped using Kaiser's Glycerin Jelly. Sections were imaged through a Zeiss Axio Observer Z1 inverted light microscope with ZEN imaging software, at × 10 and × 40 magnification. Images were captured at 88,000 dpi using the AxioCam 506 color digital camera.

### Triglycerides in blood and liver

To quantify changes in triglycerides concentrations were measured in blood and liver samples of 10 months old mice using a Triglyceride Colorimetric Assay Kit (Cat. # 10010303, Cayman Chemical, Ann Arbor MI). Ten biological replicates (one pup per litter) were analyzed per exposure group. Each biological replicate was analyzed in triplicate.

### Reanalysis of published transcriptional datasets

Changes in liver gene expression for many individual genes, as well as for groups of metabolic genes, which were observed in this current study were opposite in direction to the changes reported in our previous study (18). We hypothesize that differences in exposure protocols may have been responsible for the opposite effects in the direction of change of the gene expression in livers of exposed animals. To test this hypothesis we analyzed changes in expression of ribosomal genes, a group of genes that is most sensitive to PBDE exposure according to our current and previously published data (18), in transcriptomic datasets obtained from toxicological experiments with PBDE. The search for transcriptomic datasets was completed on June 1, 2017. Specifically, we searched Gene Expression Omnibus (GEO, http://www.ncbi.nlm.nih.gov/geo/) and ArrayExpress (https://www.ebi.ac.uk/arrayexpress/) genomic data depositories using the following key words: *PBDE, BDE, polybrominated, diphenyl ether, and flame retardant*. We also ran a search in the PubMed (http://www.ncbi.nlm.nih.gov/pubmedn) using a combination of the words *PBDE, BDE, and polybrominated diphenyl ether* with one of the following: *gene expression, transcriptome, microarray, RNA-seq, and genomic*. All selected papers were then checked for the presence of all-genome gene expression analysis and, if positive, for links to the original gene expression data. As a result of this search, we identified transcriptomic datasets produced by our research group (18, 43, 44) and another research group (45). Experimental designs of these studies are summarized in Table 2. To address changes in expression of ribosomal genes we used GSEA with the “KEGG Ribosome” gene set. This gene set includes 88 human genes of ribosomal proteins and RNA, and is curated by Kyoto Encyclopedia of Genes and Genomes (KEGG, http://www.genome.jp/kegg/).

**Table 2:** Gene set enrichment analysis (GSEA) of ribosome dataset in transcriptomic studies of PBDE effect in rodents and details of experimental design of these studies.

**Model**	**Sex**	**Tissue**	**Exposure chemical**	**Daily dose, mg/kg/body weight**	**Exposure duration (ED–embryonic day, (PND–postnatal day)**	**PND for outcome**	**Gene expression analysis approach**	Normalized enrichment score/Nominal *p*-value KEGG^*^ribosome	**Source**
Rat	Male	liver	DE-71^**^	50	ED6-PND22	22	Affymetrix Rat Genome 230 2.0 Array	−2.14/0.000	(45)
Rat	Female	liver	DE-71^**^	50	ED6-PND22	22	Affymetrix Rat Genome 230 2.0 Array	−1.98/0.000	(45)
Rat	Male	liver	DE-71^**^	50	ED6-PND91	91	Affymetrix Rat Genome 230 2.0 Array	−1.97/0.000	(45)
Rat	Male	liver	BDE-47	0.2	ED15-PND21	27	Illumina BeadChips RatRef-12	1.39/0.034	(43)
Rat	Female	brain frontal lobes	BDE-47	0.2	ED15-PND21	41	Illumina BeadChips RatRef-12	1.40/0.033	(44)
CD1 mice	Male	liver	BDE-47	0.2	ED8-PND21	21	Illumina TruSeq RNA-Seq	1.60/0.006	(18)
CD1 mice	Male	liver	BDE-47	0.2	ED8-PND21	140	Illumina TruSeq RNA-Seq	3.21/0.000	(18)
CD1 mice	Male	liver	BDE-47	1	ED8-ED21	300	Illumina TruSeq RNA-Seq	−2.49/0.000	Current study
CD1 mice	Male	liver	BDE-47	1	PND1-PND21	300	Illumina TruSeq RNA-Seq	−1.59/0.019	Current study

* *KEGG, Kyoto Encyclopedia of Genes and Genomes*.

** *DE-71 is a commercial mix of PBDE, which includes primarily the tetra- through penta-PBDEs and a small component of hexa-BDE*.

### Statistical analysis

All statistical analyses were performed using SPSS Statistics 22 software. ANOVA with subsequent Dunnett's test were used to compare body weights and triglyceride values in blood and liver between exposed and control groups. *T*-test was used to compare western blot densitometry for CD36 protein expression between control and each exposed group. Correlation coefficient was calculated to determine reproducibility of RNA-seq results using RT-qPCR for selected genes and to compare effects of pre- and neonatal exposures on gene expression.

## Results

We found no significant relationship between litter size and exposure to BDE-47, with the number of pups varying from 9 to 15 per litter. On PND300, body weights of male animals were 58.5 ± 1.9 g in the control group, 52.5 ± 1.4 g in the prenatally exposed group (*p* = 0.02), and 56.6 ± 1.9 g in the neonatally exposed group (*p* = 0.09). All data are mean ± SE and *p*-values are for comparison with controls.

### Pre-and neonatal exposures to BDE-47 induce similar transcriptomic changes in livers of 10 months old mice

**Figure 1:**
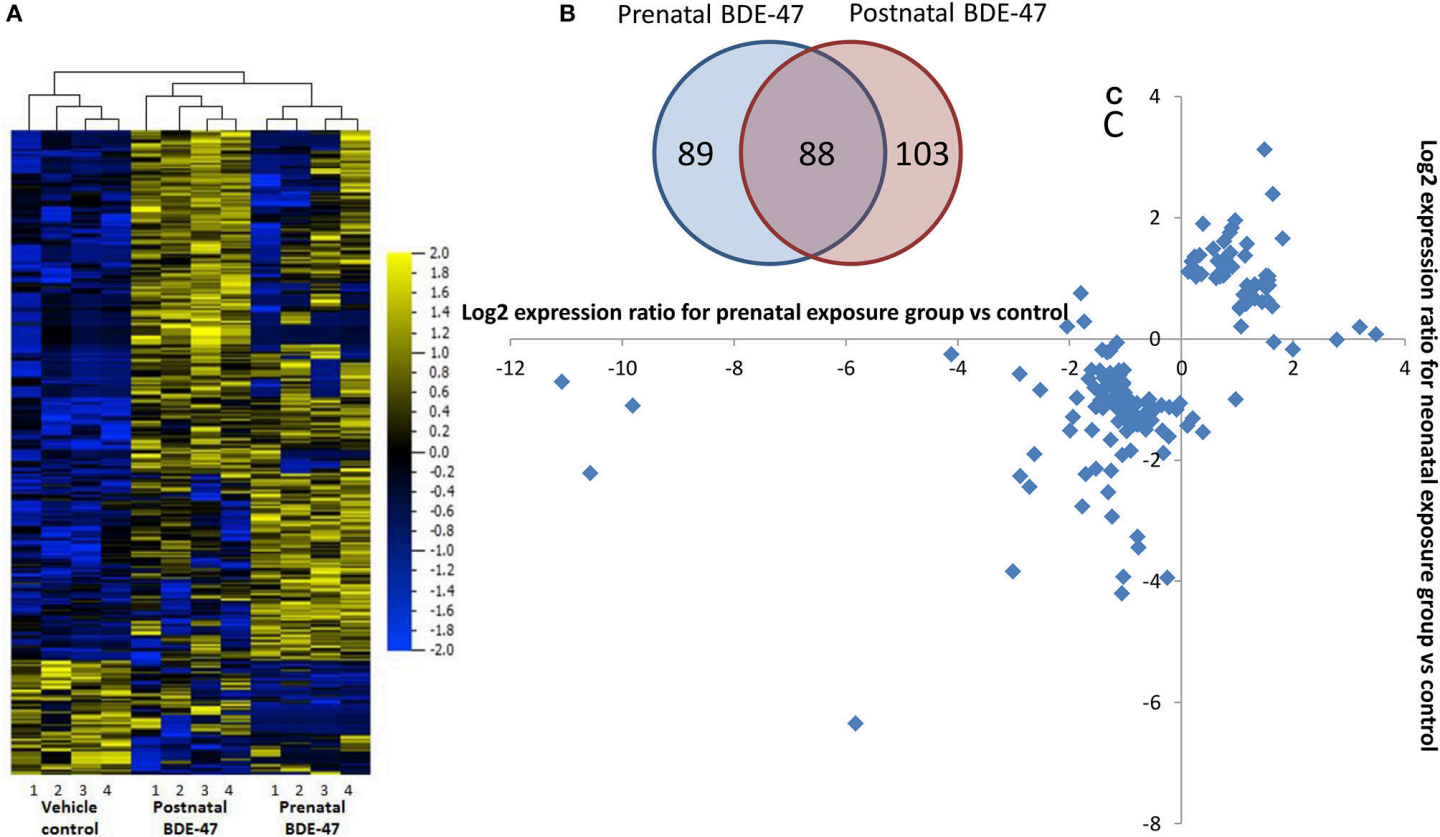
Changes in liver gene expression in 10 months old mice are highly overlapping in groups exposed prenatally only and neonatally only to 1 mg/kg/body weight BDE-47: **(A)**–overlap in lists of significantly (FDR *q* ≤ 0.05) 2-fold differentially expressed genes; **(B)**–heat-map and unsupervised hierarchical clustering based on merged list 280 genes significantly (FDR *q* ≤ 0.05) 2-fold differentially expressed in pre- and/or postnatally exposed groups; **(C)**–changes in expression of non-overlapping genes (89 + 103 genes) in pre- and neonatal exposure groups, for majority of genes direction of change is the same in both groups.

We analyzed using an RNA-seq approach transcriptional changes in liver tissue of 10 months old male mice. Sequencing was completed with an average of 45 million reads per sample and more than 90% of the reads aligned to the reference genome. After filtering out transcripts that did not correspond to any of known identifiers (genes, non-coding RNAs) and those that had LogFPKM ≤ 0 in both conditions (control and exposed), lists of genes with differential expression values were generated for both exposure groups. These lists consisted of 10,962 transcripts for the prenatal exposure group and 11,035 transcripts for the neonatal exposure group. Short lists of the significantly differentially expressed genes were generated using cut-off thresholds of 2-fold change in expression and a false discovery rate (FDR) *q* ≤ 0.05. RNA-seq results were confirmed by RT-qPCR for select genes (Table 1). Correlation coefficients between values of gene expression change measured by RNA-seq and RT-qPCR were 0.98 in both exposure groups. Short lists of genes that were differentially expressed following prenatal or neonatal exposure consisted of 176 and 191 genes, respectively. These lists overlapped for 88 genes (Figure 1A). Correlation coefficient between values of gene expression change in the merged list of 279 genes significantly altered in either exposure group was 0.87. Most of these genes that did not overlap were still altered in the same direction in both exposure groups although with lower significance and fold change (Figures 1B,C). In fact only 10 out of 191 non-overlapping genes were altered in different directions and correlation coefficient was 0.52 for the merged list of non-overlapping genes between exposure groups.

### Developmental exposure to BDE-47 alters blood/liver balance of triglycerides and leads to lipid accumulation in livers of 10 months old mice

**Figure 2:**
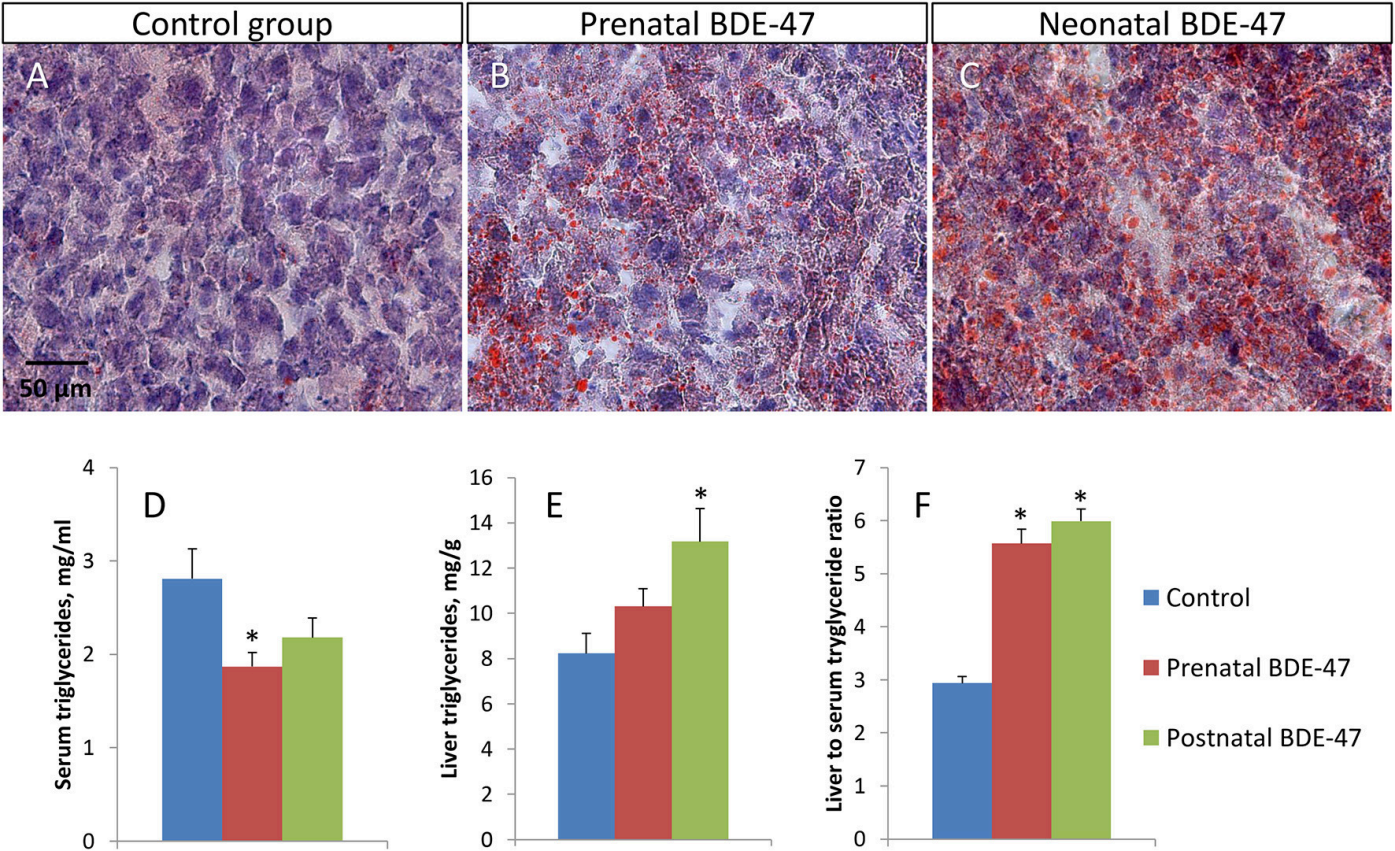
Developmental exposure to BDE-47 results in altered triglyceride concentrations in hepatocytes and blood of 10 months old mice. **(A–C)**–representative liver tissue sections stained with Oil Red O and Harris' hematoxylin (×400): **(A)**–control group, **(B)**–prenatal exposure group, **(C)**–neonatal exposure group. Lipid droplets are stained in orange. **(D,E)**–triglyceride concentrations in blood and liver, respectively. **(F)**–liver to serum triglyceride ratio. All data are Mean ± SE, *n* = 10/exposure group, ANOVA followed by Dunnett's test, *p < 0.05 when compared with control group.

We analyzed concentrations of triglycerides in serum and in livers of exposed and control animals. We also used Oil Red O staining of liver sections to visualize triglyceride droplets. In both exposed groups, liver tissue had a higher number of lipid droplets in hepatocytes (Figures 2A,B). Triglycerides concentrations in serum were decreased in both exposure groups compared to the control group (2.81 ± 0.32 mg/ml in the control group; 1.87 ± 0.15 mg/ml, *p* = 0.019 in the prenatal exposure group; and 2.18 ± 0.21 mg/ml, *p* = 0.131 in the neonatal exposure group)—Figure 2D. Concentrations of triglycerides in the liver were increased in both exposure groups (8.23 ± 0.88 mg/g in the control group; 10.31 ± 0.79 mg/g, *p* = 0.102 in the prenatal exposure group; and 13.18 ± 1.46 mg/g, *p* = 0.015 in the neonatal exposure group)—Figure 2E. The ratio of liver to serum triglyceride concentrations was significantly higher in both exposed groups (2.94 ± 0.12 in the control group; 5.57 ± 0.27, *p* = 3.3E-07 in the prenatal exposure group; and 5.99 ± 0.23, *p* = 1.3E-08 in the neonatal exposure group)—Figure 2F. All data shown are mean ± SE and *p*-values are for comparison with controls.

### Pre-and neonatal exposures to BDE-47 alters expression of genes responsible for lipid metabolism and protein biosynthesis in livers of 10 months old mice

**Figure 3:**
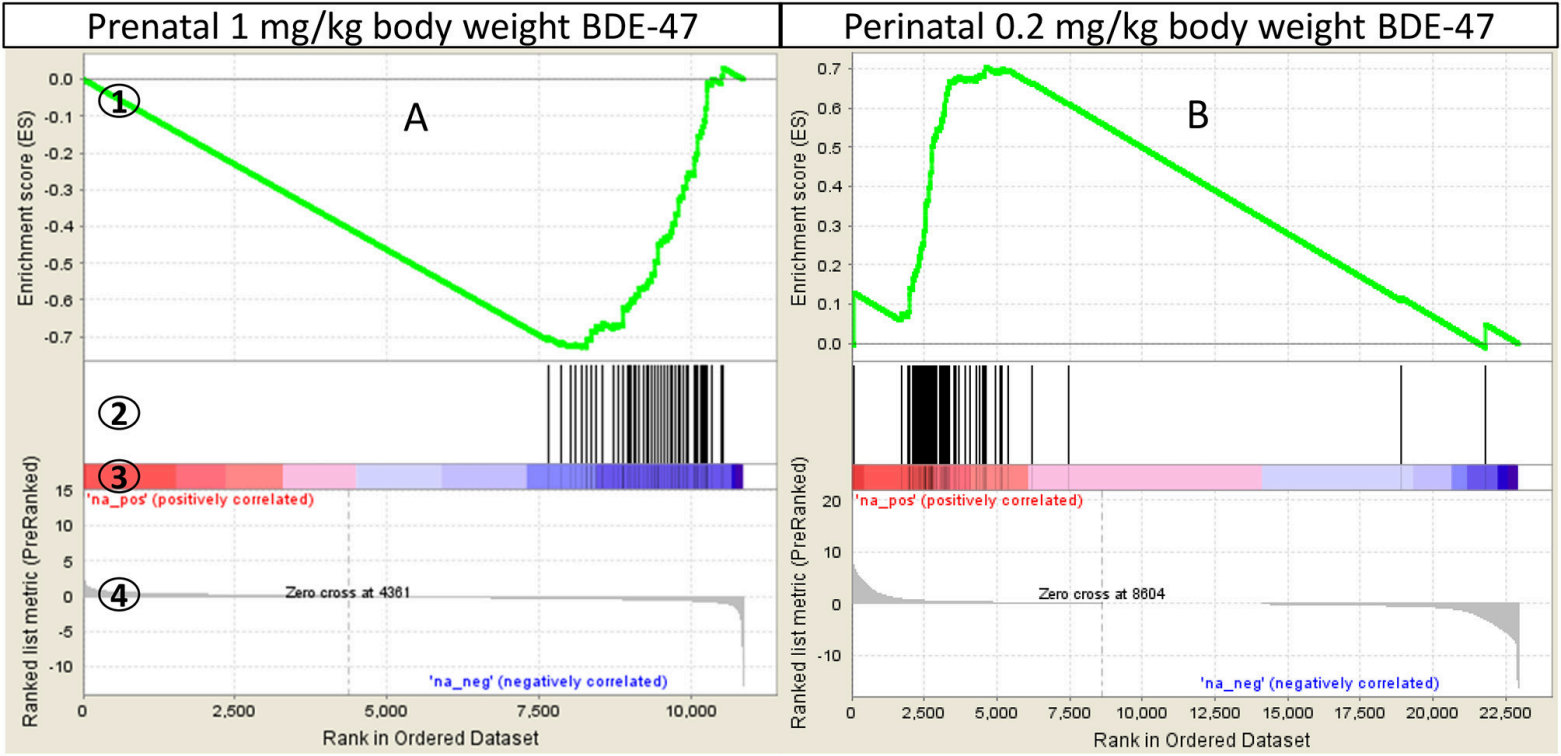
GSEA enrichment of “KEGG Ribosome” gene set in mouse liver: **(A)**–mice were exposed prenatally to 1 mg/kg body weight BDE-47 and gene expression analyzed on PND300; **(B)**–mice were exposed perinatally to 0.2 mg/kg body weight BDE-47 and gene expression analyzed on PND140 (18). GSEA plot legend: 1-running enrichment score for the gene set; 2-vertical lines show where the members of the gene set appear in the ranked list of genes; 3 and 4-ranked list of differentially expressed genes from the most up-regulated (left of each plot) to the most downregulated (right of each plot). Regulation is shown in the heat-bar (3) and in the bar plot (4).

Gene sets enriched with Normalized Enrichment Score >1.6, nominal *p* ≤ 0.05 and FDR *q* ≤ 0.2 were selected for further analysis. Thus, we used a more stringent criteria than that recommended by GSEA developers (FDR *q* ≤ 0.25, http://software.broadinstitute.org/gsea/doc/) for a more exclusive focus on significantly altered biological processes. Gene sets that satisfy these criteria in at least one exposure group (prenatal or neonatal) are shown in Table 3 and Supplemental File 2. All negatively enriched gene-sets from KEGG and Reactome collections and a few from the Hallmark collection point to a suppression of mRNA processing, translation and post-translational processing of protein, such as: KEGG: Ribosome (Figure 3A) and Spliceosome; Reactome: Translation, Peptide Chain Elongation, Protein Metabolism, mRNA Metabolism and Protein Folding; Hallmark: Protein Secretion and others. The gene set of an mTORC1 pathway, which is a major pathway of protein synthesis control, was also negatively enriched (Hallmark: mTORC1 Signaling). Gene sets from Hallmark also include negatively enriched gene-sets that are related to cellular stress (Unfolded Protein Response, DNA Repair), cell cycle progression, apoptosis and cellular transformation (MYC Targets V1 and V2), and immune response (Interferon Gamma and Interferon Alpha Response). Significant Hallmark gene-sets relevant for lipid metabolism include negatively enriched Cholesterol Homeostasis gene set and positively enriched Bile Acid Metabolism gene set. Cholesterol Homeostasis gene set was negatively enriched partly due to decreased expression of genes encoding proteins responsible for lipid transport into cells (*Plscr1, Fabp5, Lpl*) and genes encoding enzymes of cholesterol biosynthesis (*Sqle, Hmgcs1, Idi1, Mvd*). Some of the latter genes encode for rate-limiting enzymes of different stages of cholesterol synthesis (*Sqle, Hmgcs1*). The positively enriched Bile Acid Metabolism gene set was due to the increased expression of many genes participating in bile acid biosynthesis, including many key enzymes of the pathway (*Cyp7a1, Cyp46a1, Cyp27a1, Cyp8b1, Akr1d1, Slc27a2, Slc27a5, Amacr, Hsd17b4*).

**Table 3:** Top GSEA enriched gene-sets in livers of 10 months old mice exposed pre- or neonatally to BDE-47.

**Gene set**	**Prenatal exposure**			**Neonatal exposure**		
	**NES**	**NOM *p*-val**	**FDR *q*-val**	**NES**	**NOM *p*-val**	**FDR *q*-val**
**HALLMARK COLLECTION OF GENE-SETS**						
Unfolded protein response	−**2.06**	**0.000**	**0.003**	−**1.98**	**0.000**	**0.003**
MYC targets V2	−**2.04**	**0.000**	**0.002**	−**1.81**	**0.006**	**0.017**
MYC targets V1	−**2.01**	**0.000**	**0.002**	−**2.00**	**0.000**	**0.004**
DNA repair	−**1.93**	**0.000**	**0.006**	−**1.62**	**0.011**	**0.054**
Cholesterol homeostasis	−**1.66**	**0.010**	**0.060**	−**1.80**	**0.008**	**0.018**
MTORC1 signaling	−**1.61**	**0.005**	**0.074**	−**1.64**	**0.004**	**0.052**
Interferon gamma response	−1.39	0.050	0.234	−**2.04**	**0.000**	**0.006**
Interferon alpha response	−1.38	0.086	0.205	−**1.84**	**0.001**	**0.016**
Protein secretion	−1.22	0.181	0.365	−**1.65**	**0.007**	**0.052**
Bile acid metabolism	1.62	0.020	0.409	**2.16**	**0.000**	**0.056**
**KEGG COLLECTION OF GENE-SETS**						
Ribosome	−**2.49**	**0.000**	**0.000**	−1.59	0.019	0.266
Spliceosome	−**1.92**	**0.000**	**0.087**	−1.86	0.000	0.187
RNA polymerase	−**1.90**	**0.007**	**0.097**	−1.48	0.072	0.302
Protein export	−1.85	0.014	0.107	−**2.04**	**0.002**	**0.085**
**REACTOME COLLECTION OF GENE-SETS**						
Translation	−**2.62**	**0.000**	**0.000**	−1.87	0.000	0.187
SRP dependent cotranslational protein targeting to membrane	−**2.56**	**0.000**	**0.000**	−1.81	0.000	0.196
Influenza viral RNA transcription and replication	−**2.53**	**0.000**	**0.000**	−1.64	0.004	0.247
3 UTR mediated translational regulation	−**2.51**	**0.000**	**0.000**	−1.62	0.009	0.256
Peptide chain elongation	−**2.46**	**0.000**	**0.000**	−1.51	0.031	0.292
Influenza life cycle	−**2.40**	**0.000**	**0.000**	−1.64	0.009	0.247
Nonsense mediated decay enhanced by the exon junction complex	−**2.33**	**0.000**	**0.000**	−1.47	0.034	0.301
Metabolism of proteins	−**2.31**	**0.000**	**0.000**	−1.81	0.000	0.199
Activation of the mRNA upon binding of the cap binding complex and EIFS and subsequent binding to 43S	−**2.29**	**0.000**	**0.001**	−1.52	0.035	0.290
Formation of the ternary complex and subsequently the 43S complex	−**2.25**	**0.000**	**0.001**	−1.54	0.036	0.279
Metabolism of mRNA	−**2.20**	**0.000**	**0.004**	−1.46	0.016	0.307
Metabolism of RNA	−**2.18**	**0.000**	**0.004**	−1.57	0.003	0.271
mRNA splicing minor pathway	−**2.11**	**0.000**	**0.014**	−1.74	0.015	0.224
Protein folding	−**1.95**	**0.000**	**0.072**	−1.77	0.009	0.213
mRNA splicing	−**1.93**	**0.000**	**0.081**	−1.75	0.003	0.219
Activation of chaperone genes by XBP1S	−**1.89**	**0.006**	**0.100**	−**2.03**	**0.000**	**0.094**

*NES, normalized enrichment score; NOM p, nominal p-value; FDR q, false discovery q-value. All numbers for gene sets enriched with nominal p ≤ 0.05 and FDR q ≤ 0.2 are shown in bold*.

DAVID Functional Annotation Clustering analysis was done separately for upregulated and downregulated genes from the shortlists of significantly altered genes in the prenatally and postnatally exposed groups. The most enriched clusters are shown in the Table 4. The “*Cytochrome P450*” cluster was positively enriched in the postnatal exposure group due to the upregulation of cytochrome genes, largely in family 2. The “*Transmembrane and transmembrane transport*” cluster was positively enriched in both exposure groups and included up-regulated solute carrier genes, ABC transporters, oxysterol binding proteins, desmogleins, *Cd36* and others. Negatively enriched categories include “*endoplasmic reticulum”* (postnatal exposure group only), “a*cute-phase response, HDL, fatty acid binding,” “metallothionein,”* and “*extracellular, glycoprotein.”* The analysis of annotations of genes in the cluster “*endoplasmic reticulum”* showed their functional heterogeneity. Most of these genes grouped in one of three functions: vesicle transport to Golgi (*Ehd4, Golt1b, Lrrc59, Sec23b, Tmed3, Tmed9*), response to ER-stress (*Creb3l2, Creld2, Dnajb9, Hyou1, Manf*, *Sdf2l1*), and fatty acid metabolism (*Acsl4, Aldh3a2, Elovl3, Elovl7, Fndcb3, Insig2, Mrap, Mrap2*). The “*Acute-phase response, HDL, fatty acid binding*” and the “*extracellular, glycoprotein”* clusters were enriched due mostly to the decreased expression of genes encoding serum lipid-binding proteins. This includes orosomucoid genes (*Orm1, Orm2, and Orm3*), which are carriers of basic and neutrally charged lipophilic compounds, high-density lipoprotein (HDL) associated apolipoproteins (*Saa1, Saa2 and Saa3*), chylomicron associated apolipoprotein A-IV (*Apoa4*) and fatty acid binding proteins (*Fabp4 and Fabp5*). The “*Metallothionein*” cluster was negatively enriched in both exposure groups due to the downregulation of three metallothionein proteins (*Mt1, Mt2, and Mt4*).

**Table 4:** Top enriched DAVID clusters in livers of 10 months old mice exposed prenatally or neonatally to BDE-47.

**Cluster of enriched terms**	**Prenatal exposure group**		**Neonatal exposure group**	
	ES^*^	**Benjamini *p***	ES^*^	**Benjamini *p***
**POSITIVELY REGULATED FUNCTIONS**				
Cytochrome P450	3.13	6.0E-1–2.0E-4	6.14	5.6E-1–1.8E-9
Transmembrane, transmembrane transport			2.22	9.8E-1–5.2E-4
**NEGATIVELY REGULATED FUNCTIONS**				
Endoplasmic reticulum			4.82	5.8E-3–4.3E-5
Acute-phase response, HDL, fatty acid binding	1.51	9.9E-1–3.2E-1	3.89	9.8E-1–9.9E-5
Metallothionein	3.62	7.5E-1–1.6E-2	3.50	3.1E-1–7.9E-3
Extracellular, glycoprotein	1.86	8.1E-1–1.5E-1	3.43	1.0E0–1.1E-6

* *ES, enrichment score*.

**Figure 4:**
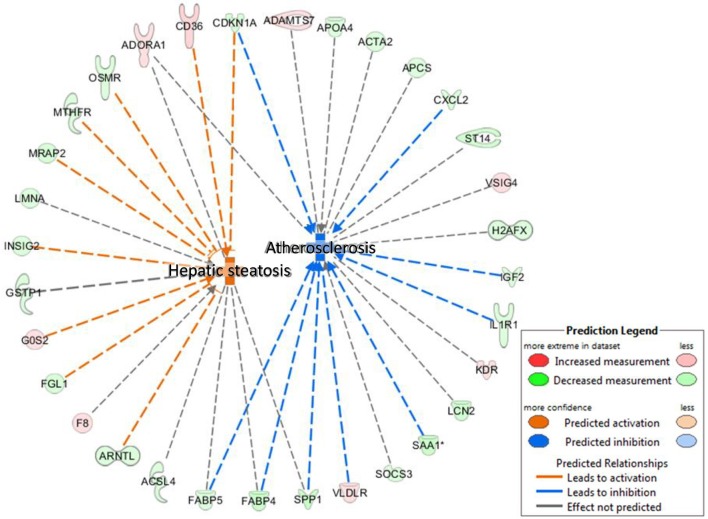
IPA Disease and BioFunctions analysis: hepatic steatosis and atherosclerosis—significantly positively and negatively enriched categories in livers of 10 months old mice developmentally exposed to 1 mg/kg body weight BDE-47.

We performed an IPA Disease and BioFunctions analysis with default settings. Initially, this analysis was performed for three lists of differentially regulated genes: genes altered in animals exposed to BDE-47 *in utero*, genes altered in animals exposed to BDE-47 neonatally, and a merged list of genes regulated in both groups. The results of this analysis were very similar for all three gene lists, therefore we present here only data for the merged list of genes (see Supplemental File 3 for details). The “*Concentration of lipid*” was the most significantly enriched category (*p* = 7.1E-08). The “*Hepatic steatosis*” cluster was one of the most significantly enriched categories with high predicted activation (*p* = 4.69E-07, activation *z*-score = 2.89), while “*Atherosclerosis*” was among the most significant categories with predicted suppression (*p* = 5.25E-06, activation *z*-score = −2.35; Figure 4).

**Figure 5:**
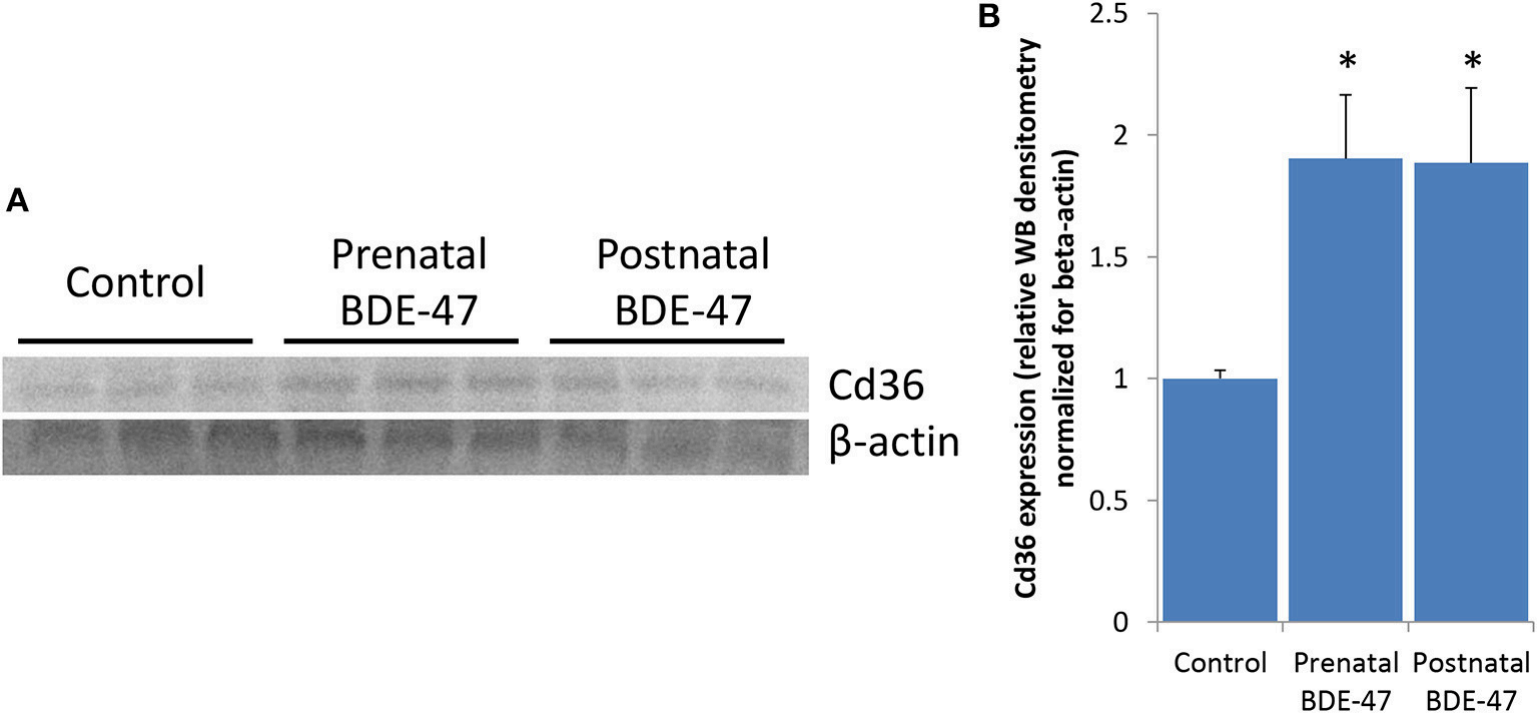
Expression of Cd36 protein is increased in livers of 10 months old mice exposure to BDE-47 during prenatal or neonatal development: **(A)**–expression of Cd36 measured by western blot; **(B)**–quantification of Cd36 protein expression (Mean ± SE, *n* = 3/exposure group, *p*-value is for *T*-test).

To explore what mechanisms of liver lipid metabolism altered by BDE-47 may be responsible for increased triglycerides in livers of exposed animals we analyzed changes in expression of key enzymes of lipid trafficking, de-novo synthesis and/or disposal (Supplemental File 1). *Cd36* is the only gene form this list that was significantly altered in both exposed groups, with 2.3- and 5.6-fold increase in expression in prenatally and postnatally exposed animals, respectively. Higher magnitude of change of *Cd36* in postnatally exposed animals correspond to higher concentrations of triglycerides in their livers. Expression of Cd36 protein was also significantly increased in liver of animals from both exposed groups as confirmed by western blot data (Figure 5).

### Low and moderate/high exposures to PBDE regulate expression of metabolic genes in opposite directions

In both the current study, and our previous study (18), ribosomal genes represented one of the most sensitive groups of genes that underwent coordinated changes in expression following PBDE exposure. However, changes in expression of ribosomal genes had an opposite direction in these studies. Similarly, many other important metabolic genes, including Cd36 were significantly differentially expressed in both studies but the direction of change was different. To test whether variables in the experimental protocol may have contributed to the observed opposite effects in expression of metabolic genes, we analyzed changes in the expression of ribosomal genes in transcriptomic datasets obtained from previously published toxicological experiments with PBDE (18, 43–45) as described in Reanalysis of Published Transcriptional Datasets. The results of this analysis are summarized in Table 2. In short, (i) the ribosomal gene set was enriched in GSEA analysis with nominal *p*-value < 0.05 in all reanalyzed datasets, supporting our observation that coordinated changes in ribosomal gene expression represent a molecular signature of PBDE exposure. (ii) In the studies that used rats and mice, similar exposures produced similar changes in ribosomal gene expression. For example perinatal exposure to 0.2 mg/kg BW BDE-47 in Wistar rats and in CD-1 mice significantly positively enriched ribosomal dataset in liver tissue (18, 43). (iii) One study focused on gene expression in the brain frontal lobes (44) and it showed similar changes in ribosomal gene expression as studies that used similar exposures to analyze gene expression in liver tissue (43, 44). (iv) Finally, the opposite effects were observed in studies using 0.2 mg/kg BW BDE-47 (18, 43, 44) and studies using 1 mg/kg BW BDE-47 (current study) or 50 mg/kg BW DE-71 (45)—a commercial mix of PBDE, which includes primarily the tetra- through penta- PBDEs and a small component of hexa-BDE.

## Discussion

We have found that both prenatal and neonatal exposure to 1 mg/kg body weight of BDE-47 result in similar changes in expression of metabolic genes in 10 months old mouse liver, suggesting permanent reprogramming of the liver metabolism in both exposure groups. This reprogramming includes increased expression of *Cd36* and other lipid transporters and decreased expression of serum lipid-binding proteins which is a likely mechanistic explanation of the observed shift in the balance of triglycerides toward their reduced concentrations in the blood and increased concentrations in the liver. Long-lasting or permanent shift in liver metabolism, leading to excessive accumulation of triglycerides in the liver, may be a risk factor for the development of liver steatohepatitis—the most common form of chronic liver disease among adults and children (46, 47) with 33% to 88% prevalence (48–51). Liver steatohepatitis is a risk factor for type 2 diabetes, dyslipidemia, hypertension, cardiovascular and kidney disease, liver cirrhosis, hepatocellular carcinoma, and mortality (52–57).

### Relevance of our dosing paradigm

In our previous study (58), exposure of pregnant rats to 0.2 mg/kg body weight of BDE-47 resulted in 234.3 ng BDE-47/g lipid in adipose tissue of dams and 1,054.7 ng BDE-47/g lipid in adipose tissue of pups. These concentrations are comparable with those of the North American human population (mean concentration in adipose tissue of adult subjects = 399 ng/g lipids) (2). Given that the rate of BDE-47 elimination is around 10 times higher in mice than in rats (59, 60) the exposure used in this study may also be relevant to that of the North American human population. To further increase the relevance of our exposure paradigm to human exposures, we dosed animals during pre- or neonatal periods since human PBDE exposure peaks during the perinatal period of life due to the transport of PBDE *via* cord blood and breast milk (19, 20, 22), higher rates of dust ingestion (21), and higher food intake per kilogram of body weight in toddlers (22).

### Long lasting programming of liver metabolism

In our previous studies, we have demonstrated that perinatal exposures to low doses (0.2 mg/kg body weight) of BDE-47 may produce long lasting changes in gene expression in rodent livers. In our rat, study expression of many metabolic genes including genes of cholesterol metabolism and ribosomal proteins, were significantly altered 1 week after the last day of exposure (43). Blood cholesterol was also increased in exposed rats. In a recent study with CD-1 mice similar changes in liver gene expression were observed on postnatal day 140, ~4 months after the last day of exposure (18). Exposed mice had significantly increased blood triglycerides. These findings indicate that metabolic reprogramming of liver by developmental exposure to PBDE is likely permanent. A long-lasting change in liver lipid metabolism by PBDEs is also supported by the current study as it demonstrates altered expression of metabolic genes in liver and increased accumulation of liver triglycerides 10 months after the last day of exposure. A review of extant toxicological evidence linking exposures to different chemicals with hepatic steatosis and steatohepatitis (27) did not list any evidence of liver lipid accumulation in adult organism resulting from developmental exposure. Thus, developmental programming of liver metabolism described in this study is a novel potential risk factor for fatty liver diseases development.

### Sensitive exposure windows

In previous studies, the perinatal window of exposure, spanning from part of gestation and postnatal development until weaning, was used to study programming effects of PBDE for the liver metabolism (18, 43, 61). In the current study, we aimed to narrow down the sensitive developmental window of liver programming by using prenatal-only and postnatal-only exposures. Surprisingly, both exposed groups developed very similar shift in expression of metabolic genes including upregulation of lipid transporter *Cd36* and suppression of genes encoding ribosomal proteins in the liver. Both exposure groups also had similar shifts in the blood-liver balance of triglycerides, including decreased serum triglycerides and increased liver triglycerides, although triglycerides accumulation in the liver was more pronounced in the neonatal exposure group. Thus, our results demonstrate that the sensitive developmental window of the liver metabolism reprogramming by BDE-47 spans across pre- and neonatal periods of development, i.e., likely corresponds to the period of hepatocyte maturation, which starts at gestation day 18.5 and covers postnatal development at least till postnatal day 45 (62–64). Postnatal exposure used in our experiment covers longer period of hepatocyte maturation than prenatal exposure, which only interferes with the beginning of this process. That fact may explain higher accumulation of triglycerides in the livers of postnatally exposed animals. It should be mentioned that BDE-47 that accumulated in the bodies of fetuses in the prenatal exposure group was not fully eliminated from the organisms by the time of delivery and could affect some molecular mechanisms during the neonatal period, resulting in similar effects in both exposure groups. Given that the half-life of BDE-47 in mice is around 3–4 days (59, 60) we assume that “carry over” of BDE-47 from prenatal life to postnatal life in the prenatal exposure group could have had health consequences only during first few days of neonatal life.

### Molecular mechanisms underlying altered liver/blood balance of triglycerides

It was shown previously that major process that contributes to lipid accumulation in liver is fatty acids uptake by hepatocytes (39, 65, 66), although de novo lipogenesis, triglyceride synthesis, as well as disposal of fatty acids in a form of oxidation and/or VLDL secretion may contribute to triglyceride concentrations in liver. To explore, perturbation of which process may be responsible for the observed accumulation of triglycerides in our study, we analyzed changes in expression of the key enzymes (Supplemental File 1) involved in lipid uptake, biosynthesis and disposal. Increased expression of *Cd36* was the only enzyme from this list that was significantly upregulated in both exposed groups at the level of gene expression. Upregulation of *Cd36* was also confirmed at the level of protein expression, suggesting that this change may be the major causative factor responsible for the altered balance of the liver/blood triglycerides. This hypothesis is additionally supported by the concordance in the magnitude of *Cd36* expression and triglycerides concentrations in livers of exposed animals, so that higher upregulation of *Cd36* in postnatally exposed animals (5.7-fold) is concordant with higher increase in concentration of triglycerides (168%) in this group as compared with smaller change in *Cd36* expression (2.3-fold) and triglyceride concentration (125%) in prenatal exposure group. Previous research demonstrates that overexpression of *Cd36* may significantly increase fatty acid uptake in liver. Cd36 upregulation induced by *in vivo* adenoviral delivery of the gene to the livers of mice was sufficient to increased hepatic triglyceride storage and dyslipidemia in mice (67). *Cd36* expression is increased in humans with NAFLD (68–70). Decreased rate of VLDL secretion may also contribute to increased triglycerides in the livers and decreased in blood of exposed mice. Genes of major enzymes involved in VLDL secretion (*Apob, Mttp, Ces1d, Ces1g, Ces3a, Cideb*) were not altered significantly, however many genes responsible for VLDL trafficking between endoplasmic reticulum (ER) and Golgi apparatus (*Ehd4, Golt1b, Lrrc59, Sec23b, Tmed3*, and *Tmed9*) and genes involved in binding of lipids in blood (*Orm1, Orm2, Orm3, Saa1, Saa2, Saa3, Apoa4, Fabp4, and Fabp5*) were significantly downregulated. Triglycerides and other lipid components are synthesized in smooth ER and combine with apolipoprotein B at the junction of smooth and rough ER. The nascent particles are then transferred to the Golgi apparatus for further processing and secretory VLDL vesicles are subsequently released from the Golgi (39, 71, 72). Thus, interruption of ER-Golgi trafficking may slow down VLDL secretion by hepatocytes. Decreased expression of genes encoding serum lipid-binding proteins may also contribute to decreased concentration of triglycerides in blood.

### Opposite effects of different dosing protocols

As previously mentioned our recent study showed that CD-1 mice exposed perinatally to 0.2 mg/kg body weight BDE-47 (Table 2) had significantly upregulated genes of ribosomal proteins (Figure 3B), significantly downregulated expression of *Cd36*, and almost 2-fold increased concentration of triglycerides in blood (18). Other studies have shown similar effects of genes of ribosomal proteins being upregulated in the livers and brain frontal lobes of rats exposed to the same concentration of BDE-47 perinatally (43, 44). However, experimental data published by Dunnick and others (45) demonstrate opposite effect, genes of ribosomal proteins was the most negatively enriched gene-set. Dunnick and others exposed rats to 50 mg/kg body weight of DE-71, a commercial mix of PBDE, from embryonic day 6 continuously throughout the animals' lifetime until euthanasia (PND22 or PND91). Given that a significant shift (although in different directions) in expression of ribosomal genes was found in all transcriptomic studies of PBDE effects (Table 2), we used this “molecular signature” of PBDE to determine whether differences in experimental design were responsible for opposite outcomes. Existing studies used different animal models, timing of exposure, exposure dose, timing of outcome measurement, time interval between exposure and outcome measurement and chemical compound (BDE-47 or DE-71). Interestingly, same dose of BDE-47 produced similar changes in ribosomal gene expression in different tissues (43, 44), in different models (18, 43, 44) and in tissues collected at different time-intervals after exposure (18, 43, 44). However, the same compound used at higher dose produced opposite effect in the current study. This effect was similar to the one induced by high dose of PBDE mix (45). Thus, data presented in Table 2 suggest, that the critical parameter responsible for the direction of change of ribosomal gene expression is likely the dose of the compound. Future research should include targeted dose-response experiments to test this hypothesis.

### Concern for human health

As discussed in the introduction, approximately one-fifth of the US population (~60 million) experienced their perinatal development when concentrations of PBDE plateaued at their highest environmental levels (17, 18). If the PBDE-induced programming effects described in the current study are seen in humans, we may anticipate long-term adverse consequences of developmental exposures to PBDE. Our study demonstrates that different exposure scenarios in rodents may result in the shift in liver-blood lipid balance in either direction. Our studies indicate, that smaller doses suppress lipid uptake by the liver, resulting in triglyceride accumulation in serum. Higher doses of PBDE induce lipid uptake and accumulation in the liver. Importantly, both smaller and higher doses discussed here are relevant for the general population. Fatty changes in liver were previously shown in juvenile mice exposed for 28 days to 0.45 mg/kg body weight of BDE-47 (73). Liver steatosis associated with increased expression of *Cd36* was recently shown in mice exposed to low doses of BDE-47 perinatally and further kept on high fat diet for 14 weeks (74). Liver fatty degeneration was also demonstrated in adult rats exposed to high dose (2,000 mg/kg body weight) of pentaBDE (75, 76) and in prepubertal rats exposed to high doses (300 and 600 mg/kg body weight) of decaBDE (77). The link between PBDE exposures and lipid imbalance was never tested in human population studies.

Tight regulation of fatty acids (FA) uptake by the liver is the major process that contributes to a healthy balance of lipids between the blood and the liver (65, 66). Abnormal shifts in this balance in either direction may result in increased morbidity and mortality risks. An increase in the uptake of fatty acids and accumulation of triglycerides in lipid droplets results in NAFLD. NAFLD increases the risk of type 2 diabetes, dyslipidemia, hypertension, cardiovascular and kidney disease, liver cirrhosis, hepatocellular carcinoma, and mortality (52–57). Treatment options for NAFLD have very limited efficacy. On the other hand, decreased uptake of fatty acids by the liver may result in hyperlipidemia and atherosclerosis—the primary risk factors for heart attack, which is the most common cause of mortality in the developed world (29, 78–80).

### Molecular mechanisms of liver metabolism programming by PBDE

**Figure 6:**
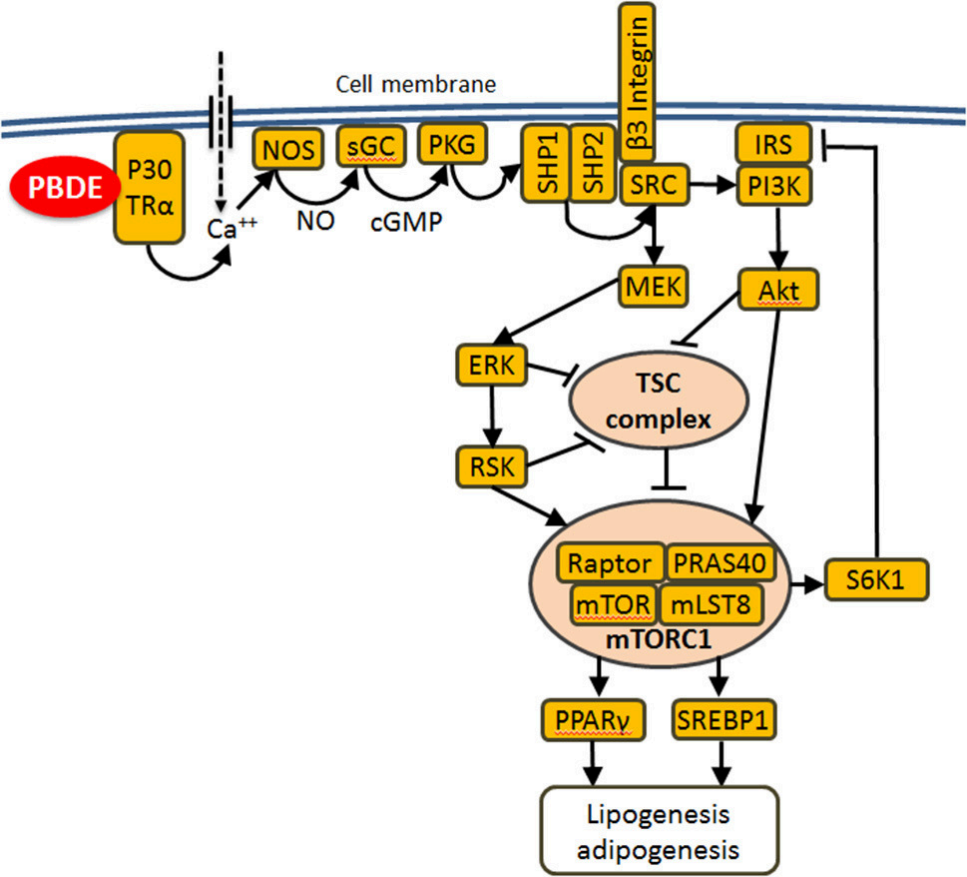
Proposed mechanism of metabolic reprograming by PBDE: PBDEs or their metabolites bind to the plasma membrane associated p30 TRα1 and induce an increase in intracellular Ca^2+^ concentration, which leads to activation of the NO-cGMP-PKGII signaling cascade and the phosphorylation and activation of the SHP1/SHP2 phosphatase complex. This complex activates SRC which in turn activates MEK-ERK and PI3K-AKT signaling. Both cascades merge on and suppress tuberous sclerosis complex (TSC), which is a potent mTORC1 suppressor. Lipogenesis and adipogenesis are regulated by mTORC1 mainly via SREBP1/2 and PPARγ transcription factors that control the expression of genes involved in fatty acid and cholesterol synthesis, lipid uptake and storage. Additionally mTORC1 directly phosphorylates/activates S6K1 which phosphorylates/suppresses IRS1 causing insulin resistance.

Molecular mechanisms of metabolic effects of PBDE remain poorly understood. Previously, we demonstrated that BDE-47 activates mTOR (mechanistic target of rapamycin) signaling in mouse livers and in human hepatocellular carcinoma cells (18). mTOR is a serine/threonine protein kinase that emerged over the last decade as a critical signaling node that links nutrient and energy sensing to the coordinated regulation of cellular growth and metabolism (81, 82). Thus, mTOR plays a central role in lipid homeostasis (83, 84). One of the best characterized functions of mTOR pathway consists in the regulation of ribosomal biogenesis (85, 86). In the current study “*mTORC1 signaling”* and “*ribosome”* were among the most negatively enriched categories (Table 3) supporting our hypothesis, that mTOR pathway is involved in the long-lasting response to PBDE (18). The mTOR complex one (mTORC1) integrates signals from PI3K-AKT and RAS-ERK pathways upon their activation by receptor tyrosine kinases in response to insulin and insulin-like growth factors (81, 87). A recent study demonstrated the ability of BDE-47 and BDE-85 to activate PI3K-AKT signaling (88) in a thyroid receptor alpha dependent manner. Involvement of TRα and AKT points at recently described non-genomic pathway of thyroid hormone signaling (89) in which binding of T3 to the plasma membrane-associated p30 isoform of TRα1 activates the nitric oxide (NO)/cyclic guanosine monophosphate (cGMP)/protein kinase G II (PKGII) signaling cascade and results in the phosphorylation/activation of the SHP1/SHP2 phosphatase complex. Activation of the tyrosine kinase SRC by this complex results in activation of MEK-ERK and PI3K-Akt signaling. Given that PBDEs are well-characterized thyroid disruptors (90–94), we propose the following hypothesis to link developmental PBDE exposures to long-lasting programming of liver metabolism (Figure 6): PBDEs and/or their metabolites interact directly with thyroid receptors and activate downstream PI3K-AKT and MEK-ERK signaling to induce mTORC1 activity; changes in mTORC1 activity during critical windows of liver development change liver metabolic settings and induce long-lasting alterations in lipid and other metabolism. Additional mechanistic research is needed to test this hypothesis.

## Conclusions

We have found that prenatal or neonatal exposures to an environmentally relevant dose (1 mg/kg body weight) of BDE-47 result in permanent reprogramming of the liver metabolism in both exposure groups. The similarity in responses of both exposure groups indicate that the sensitive window of liver metabolism programming spans pre- and postnatal period of development. Observed reprogramming included changes in expression of genes involved in lipid and other metabolism, which resulted in reduced concentrations of triglycerides in the blood and increased concentrations of triglycerides in the liver. Opposite directional changes in expression of many metabolic genes and levels of circulating triglycerides were observed in comparison with previous studies, which used a lower exposure dose (0.2 mg/kg body weight). Both exposure doses are relevant for the general population. If similarity of toxic effects and dose-response relationships between mice and humans will be proven in future research it will indicate that the range of existing and past environmental exposures could produce changes in lipid metabolism in both directions in the general population. The shift in blood-liver balance of lipids may be associated with health conditions, such as NAFLD and atherosclerosis. Based on this study and previously published data, we propose that metabolic effects of developmental exposures to PBDE are mediated by the mTORC1 signaling pathway. Species-specific differences in the involved pathways need to be further investigated to assess the translational relevance of our findings. Additional research is needed to test the mechanistic considerations of this study, establish dose-response relations between developmental exposures and lipid metabolism in adulthood, and elucidate developmental metabolic effects of PBDE in the general population.

## Author contributions

AS conceived, designed, and coordinated the study and participated in all steps of laboratory experiments, data analysis and drafting of the manuscript. AK conducted exposure experiment with mice, and protein expression analysis. SC conducted analysis of triglycerides. SH, SK, and MR participated in libraries preparation for RNA-seq and bioinformatic analysis. All authors contributed to the manuscript editing, they read and approved the final manuscript.

### Conflict of interest statement

The authors declare that the research was conducted in the absence of any commercial or financial relationships that could be construed as a potential conflict of interest. The reviewer LM and handling Editor declared their shared affiliation.
